# FGF21 inhibits invasion and metastasis via IL-17A-Notch in pancreatic ductal adenocarcinoma

**DOI:** 10.3389/fonc.2025.1680888

**Published:** 2025-12-04

**Authors:** Fangdan Li, Panchun Zheng, Xiongwu Li, Yaying Yang, Hangyu Zhou, Linjun Li

**Affiliations:** 1 Department of Pathology, College of Basic Medicine, Chongqing Medical University, Chongqing, China; 2 Department of Pathology, The People’s Hospital of Dazu, Chongqing, The Affiliated Dazu’s Hospital of Chongqing Medical University, Chongqing, China; 3 Clinical Pathology Laboratory of Pathology Diagnostic Center, Chongqing Medical University, Chongqing, China; 4 Department of Pathology, Chongqing General Hospital, Chongqing University, Chongqing, China; 5 Molecular Medicine Diagnostic and Testing Center, Chongqing Medical University, Chongqing, China; 6 Department of Pathology, The First Affiliated Hospital of Chongqing Medical University, Chongqing, China

**Keywords:** FGF21, pancreatic ductal adenocarcinoma, IL-17A, Notch, invasion, metastasis

## Abstract

**Background:**

Pancreatic ductal adenocarcinoma (PDAC) demonstrates characteristic histopathological features marked by abundant inflammatory cell infiltration and the formation of dense fibrous stromal capsules, which contribute to its aggressive metastatic potential. Although Fibroblast Growth Factor 21(FGF21) exhibits pancreatic anti-inflammatory properties, its expression becomes markedly downregulated in PDAC. The present research explores the inhibitory effects of FGF21 on PDAC progression within inflammatory microenvironments, with particular focus on delineating the involved signaling pathways and molecular interactions.

**Methods:**

We detected FGF21 expression in human PDAC specimens and analyzed its relationship with various clinicopathological features. For *in vitro* studies, an inflammatory microenvironment of PDAC cell lines was modeled by Lipopolysaccharide (LPS) stimulation and Transwell assays were used to detect the effects of recombinant FGF21 on invasion and migration. *In vivo*, a splenic-liver metastasis model was established in nude mice and liver metastases were monitored by imaging following intraperitoneal administration of LPS or/and FGF21. Signaling pathways of FGF21 in PDAC cell lines were identified through transcriptome sequencing and bioinformatics analysis, with key components validated by RT-PCR, Western blot and ELISA.

**Results:**

Low expression of FGF21 in PDAC patients is associated with higher TNM stages and increased rates of lymph node metastasis, vascular invasion, and tumor recurrence compared to high expression of FGF21.*In vitro* and *in vivo* studies revealed FGF21 significantly suppressed migration and invasion in cultured PDAC cell lines and reduced hepatic metastasis in murine models after LPS treatment. FGF21 downregulated key Notch signaling components (HES1, HES2, RBPJ, ZEB1, Notch1, Notch3) while concurrently attenuated Interleukin-17A (IL-17A) production. After IL-17A addition to PDAC cell lines, the inhibitory effect of FGF21 on the Notch signaling pathway was significantly reduced.

**Conclusion:**

FGF21 suppresses invasion and metastasis in PDAC by inhibiting the IL-17A-Notch signaling axis, which reveals a novel therapeutic strategy for this malignancy.

## 1 Introduction

Pancreatic Ductal Adenocarcinoma (PDAC), a highly invasive malignancy of the digestive system, predominantly occurs in individuals aged 65–75 years (1). Global population aging continues to drive its rising incidence, with the International Agency for Research on Cancer (IARC) reporting 510,600 new cases worldwide in 2022- underscoring a mounting disease burden (2). This malignancy demonstrates high lethality owing to diagnostic difficulties at early stages, aggressive progression, and limited treatment options, with a current five-year survival rate of approximately 13%-substantially lower than that of other common cancers (3). Projections indicate PDAC will become the second-leading cause of cancer mortality by 2040, exceeded only by lung cancer (4). Consequently, for this highly lethal malignancy, there is an urgent need to strengthen the exploration of its pathogenesis and diagnostic and therapeutic approaches.

Fibroblast Growth Factor 21 (FGF21), a member of the FGF family, exhibits important physiological functions including anti-inflammatory, antioxidant activities and regulation of glucose and lipid metabolism (5–7). Recent studies have shown that normal pancreatic acinar cells express high levels of FGF21 and its receptors, the FGFR1 and KLB complex, which exerts essential protective effects on acinar cells through autocrine or paracrine pathways (8). Both human studies and animal experiments of acute and chronic pancreatitis reveal that reduced pancreatic FGF21 expression strongly correlates with the occurrence and severity of pancreatic edema, necrosis, and fibrosis (9–11). Previous research by our group demonstrated that KRAS oncogene mutation significantly suppressed FGF21 expression in PDAC tissue compared to normal pancreas, via inhibition of the PPARγ pathway. Exogenous administration of recombinant FGF21 to obese KrasG12D/+ mice significantly inhibited PDAC occurrence, liver metastasis and extended survival (12). Therefore, FGF21 likely plays a critical role in PDAC pathogenesis and progression, making it a promising therapeutic target for PDAC.

Pancreatic ductal adenocarcinoma (PDAC) is histologically defined by prominent inflammatory infiltration and stromal fibrosis. The crosstalk between tumor cells and the inflammatory tumor microenvironment (ITME) critically influences disease progression by facilitating invasion, metastatic dissemination, and immune evasion (13–15). Research indicates that the ITME harbors various immune cell populations—such as tumor-associated macrophages and myeloid-derived suppressor cells—which foster immunosuppression while accelerating malignancy through the sustained release of pro-inflammatory cytokines (e.g., IL-6, TNF-α, and IL-17) and chronic activation of key signaling cascades (e.g., NF-κB and STAT3). These processes collectively reinforce tumor cell viability, trigger EMT, and stimulate angiogenesis, ultimately enhancing migratory potential and metastatic spread (16–18). Moreover, cytokine secretion from inflammation-primed tumor cells recruits further immune infiltrates and activates stromal components, dynamically reshaping the tumor niche to favor survival and aggressive growth (19). Consequently, targeting the Oncogenic positive feedback cycle linking Inflammatory microenvironment and Tumor cells represents a promising therapeutic approach for PDAC (20).

Although Fibroblast Growth Factor 21(FGF21) exhibits potent anti-inflammatory effects in pancreatic tissue, its endogenous levels are markedly decreased in pancreatic ductal adenocarcinoma specimens. Our central premise posits that therapeutic supplementation of FGF21 could potentially mitigate the invasive and metastatic progression of PDAC through modulation of the tumor microenvironment. To validate this hypothesis, our investigation will initially assess clinical-pathological relationships between FGF21 levels and critical disease markers in human PDAC samples, with particular emphasis on associations with the infiltration of immune cells and the degree of fibrosis in the inflammatory microenvironment of pancreatic cancer. Complementary experimental approaches employing both cell culture systems and animal models will then evaluate whether exogenous FGF21 supplementation inhibits the invasive and metastatic behaviors of pancreatic cancer cells in inflammatory microenvironments. To elucidate the molecular mechanisms underlying FGF21-mediated suppression of pancreatic cancer progression, we employed an integrated approach combining transcriptomic profiling with computational biology and functional molecular assays. These comprehensive findings position FGF21 as a novel candidate for targeted PDAC intervention strategies.

## 2 Materials and methods

### 2.1 Human PDAC tissue

This study utilized 72 histologically verified PDAC specimens (all were primary pancreatic ductal adenocarcinoma) obtained through surgical resection at the First Affiliated Hospital of Chongqing Medical University during the period of 2018-2020. The patient population comprised 41 male and 31 female subjects, ranging in age from 49 to 72 years (median: 63.8 years). Inclusion criteria required that participants had not undergone neoadjuvant therapy prior to surgery. Complete clinical documentation and operative reports were accessible for all study subjects. Specimen acquisition followed institutional review board approval (Chongqing Medical University Ethics Committee) and receipt of written informed consent from all participants or their legal representatives.

### 2.2 Cell lines and culture

The human PDAC cell lines, capan-1 and ASPC-1, were purchased from the American Tissue Culture Collection (ATCC, USA). The capan-1 cells were cultured in IMBM medium (ATCC, USA) supplemented with 20% fetal bovine serum (FBS, Hyclone, USA), penicillin (100U/mL, Sigma, USA), and streptomycin (100 U/mL, Sigma, USA). The ASPC-1 cells were cultured in RPMI 1640 medium (GIBCO, USA) supplemented with 10% FBS, penicillin (100U/mL, Sigma, USA), and streptomycin (100U/mL, Sigma, USA). All cells were maintained at 37°C in a humidified 5% CO2 atmosphere.

### 2.3 Spleen-liver metastasis model

Male BALB/c nude mice aged 4–6 weeks were anesthetized followed by abdominal disinfection and skin incision. After exposing the abdominal cavity, we gently exteriorized the pancreas using a saline-moistened cotton swab. A suspension of 2 × 10^6^pCAG-Luc-transfected capan-1 cells in 20 ul PBS was injected into the splenic lower pole using a 100 U insulin syringe. These mice were randomly divided into 4 groups, with 3 mice in each group. The FGF21 group received daily intraperitoneal injections of FGF21 (1 mg/kg body weight) and the control group received an equivalent volume of NS (Normal saline) daily. The LPS group received intraperitoneal injections of LPS (50μg/kg body weight), three times per week, while the FGF21+LPS group received daily intraperitoneal injections of FGF21 (1 mg/kg body weight) simultaneously with the initiation of LPS injections. At 14 days and 28 days post-injection, each nude mouse received an intraperitoneal injection of Luciferin (150 mg/kg body weight) followed by imaging using an *in vivo* imaging system for analysis.

### 2.4 H&E staining

Liver tissues of the mice were immersion-fixed in 10% neutral formalin. Specimens were dehydrated and fixed, paraffin-embedded, sectioned at 4μm thickness, and mounted on glass slides. After dewaxing in xylene and rehydration through graded ethanol series, tissue sections were stained using a hematoxylin and eosin kit (Servicebio, China) according to manufacturer’s protocol. Two independent pathologists observed tissue morphology under the microscope.

### 2.5 Immunohistochemical staining and scoring

Tissue sections were dewaxed in xylene, dehydrated through a graded ethanol series, retrieved of antigen and heat, removed endogenous peroxidase using 3% H2O2, blocked with normal goat serum, incubated with primary antibody against CD68 (ready-to-use, Maixin, China), SMA (ready-to-use, Maixin, China) and FGF21(1:200, Abcam, USA) overnight at 4°C. After washed with PBS, sections were incubated with secondary antibody at room temperature for 20 minutes, incubated with streptavidin-peroxidase for 10 minutes and washed with PBS again. The color development was achieved using DAB substrate under microscopic control followed by counterstained with hematoxylin for 30 seconds, and immunohistochemical staining scoring was independently determined by two pathologists using a double-blind method.

CD68 is used to label macrophages. The five high-power fields (×400) per slide were manually selected under Olympus BX50 microscope and CD68 positive cells in each area was then counted manually, and finally the mean value was calculated (21). FGF21 staining is localized in the cytoplasm. For each case, the staining results were scored based on the percentage of positive cells combined with the staining intensity score (12).Positive cell percentage score:<5% = 0 points, 5%-25% = 1 points, 26%-50% = 2 points, 51%-75% = 3 points, >75% = 4 points. Staining intensity score: No staining = 0 points, Light yellow = 1 points, Brownish-yellow = 2 points, Tan = 3 points. The product of two scores gives the positive level: 0 points = “-”, 1–4 points = “+”, 5–8 points = “++”, 9–12 points = “+++”.

### 2.6 Sirius Red staining and scoring

Paraffin sections routine dewaxing and rehydration were followed by staining with the prepared Weigert’s iron hematoxylin solution for 10–20 minutes. The sections were then rinsed in tap water for 5–10 minutes, followed by washing in distilled water for 1 minute. Sirius Red staining solution was applied for 1 hour, then quickly washed twice with acidic working solution and followed by two washes with water. Under a conventional optical microscope, collagen fibers appear red. The level of pancreatic collagen deposition is represented by the ratio of the average stained area of collagen fibers to the total average area of the section, which is then converted to relative collagen content (12).

### 2.7 Transwell assays

Cell migration analysis: Following trypsinization, cellular suspensions from experimental groups were prepared and quantified. For migration assessment, aliquots (200μL) containing 1×10^5 cells were introduced into the upper chamber of the small chamber, while the lower compartments received 600μL of complete growth medium. After 24-hour incubation at 37°C, migrated cells were fixed, stained, and quantified microscopically.

Cell invasion evaluation: For invasion studies, a diluted Matrigel solution was prepared at a 1:8 ratio with culture medium, and 50μL of the mixture was aliquoted into the upper chamber of each small chamber (avoid generating bubbles). Following 4–6 hours of polymerization at 37°C, the experimental procedure continued identically to the migration protocol described above, with cellular quantification performed after the incubation period.

### 2.8 Enzyme−linked immunosorbent assay

The experiment was performed according to the manufacturer’s instructions (IL-17A ELISA Kit, Bio-Legend, USA). The human IL-17A standard concentrations in the tubes are 250pg/mL, 125pg/mL, 62.5pg/mL, 31.3pg/mL, 15.6pg/mL,7.8pg/mL and 3.9pg/mL, respectively. Assay Buffer A serves as the zero standard (0pg/mL). Wash 4 times Add 50uL Assay Buffer A; Add 50uL diluted standards or samples Incubate 2h, shaking; Wash 4 times Add 100uL Detection Antibody solution Incubate 1 h, shaking; Wash 4 times Add 100uL Avidin-HRP D solution Incubate 30 min, shaking; Wash 5 times Add 100uL Substrate Solution F Incubate 30 min, in the dark; Add 100uL Stop Solution; Read absorbance at 450 nm within 30 minutes.

### 2.9 Cell transcriptome sequencing

Total RNA was isolated from the samples using TRI reagent (Takara, Japan). The concentration and purity of the RNA were determined spectrophotometrically with a Nanodrop instrument, and samples with an A260/A280 ratio between 1.8 and 2.1 were deemed suitable for further analysis. Sequencing libraries were constructed from the qualified RNA samples. Subsequently, the libraries were subjected to paired-end sequencing on an Illumina Nova Seq 6000 platform by Shanghai Yuanxin Biomedical Technology Co., Ltd.

### 2.10 RNA extraction and quantitative PCR analysis

Total RNA was isolated from the samples using TRI reagent (Takara, Japan), followed by assessment of RNA concentration and purity (The method is the same as before). Subsequently, cDNA synthesis was performed following the manufacturer’s protocol (Bio-Rad, USA). Reaction Setup for a Single cDNA Synthesis Reaction: Total volume 20ul, iScript RT Supermix 4ul, RNA template 1ug, Nuclease-free water Variable (ul). Reaction Protocol:5 min at 25°C,20min at 46°C,1min at 95°C.

For quantitative real-time PCR (qPCR), reactions were carried out in a 20μL volume containing SYBR Green PCR Master Mix (Applied Biosystems, USA) on an ABI Prism 7500 detection system (Applied Biosystems, USA). The mRNA expression levels of GAPDH serve as the internal control. Thermal cycling conditions were set according to standard protocols, and relative gene expression levels were quantified using the 2−ΔΔCt method. Primer sequences are listed in **Table 1**.

**Table 1:** Primers used in quantitative real-time PCR.

Primers		Sequence (5’→3’)
HES1 (Human)	Forward	ATCACCAAGTAGCCACAAAA
	Reverse	TCTTTGGTTTATCCGGTGTC
HES2 (Human)	Forward	CTAGGCTTTTCTGCCTAGAG
	Reverse	CAGGTGTGAAATTGATGCTC
RBPJ (Human)	Forward	TTTGTCTTTCACCGCTACC
	Reverse	TTAAGGCAGAAATCTGGTGC
ZEB1 (Human)	Forward	GGCGCAATAACGTTACAAAT
	Reverse	TAACACTGTCTGGTCTGTTG
Notch1 (Human)	Forward	AATGAGTCTGTTGTGTGTCA
	Reverse	AAAATCAACATCTTGGGACG
Notch3 (Human)	Forward	CTGGTCAAAATCCCTGTGTA
	Reverse	TACCTAGACACAGACCAACA
GAPDH(Human)	Forward	CAACCGGGAAGGAAATGAATG
	Reverse	GCCCAATACGACCAAATCAG

### 2.11 Western blot

Cellular proteins were extracted, and their concentrations were determined via the bicinchoninic acid (BCA) assay. Depending on the molecular size of the target proteins, either 10% or 12% resolving gels with 5% stacking gels were prepared for sodium dodecyl sulfate-polyacrylamide gel electrophoresis (SDS-PAGE). Following electrophoresis, separated proteins were transferred onto polyvinylidene fluoride (PVDF) membranes.

Protein bands were visualized using a Tanon 6600 chemiluminescence imaging system, and relative expression levels were quantified by optical density analysis with Image Pro Plus 6.0 software. Primary antibodies were diluted as follows: HES1 (Abcam, USA):1:500; HES2 (Abbexa, Cambridge, UK):1:1000; RBPJ (Abcam, USA):1:1000; ZEB1 (Abcam, USA):1:500; Notch1 (Abcam, USA):1:1000; Notch3 (Abcam, USA):1:1000; GAPDH (Abcam, USA): 1:1000 (loading control). Secondary antibody was diluted as follows: Goat Anti‐Rabbit IgG H&L (HRP, Abcam):1:5000.

### 2.12 Statistical analysis

Data were analyzed and plotted using GraphPad Prism 5 (Version 5.01) software. All results are expressed as the mean ± SD from at least three independent samples (*in vitro*) or from three independent animals per group (*in vivo*). Statistical differences between groups were assessed using one-way ANOVA and T-test. Chi-Square Test was used for correlation analysis. All tests were two- tailed. A *P*-value of< 0.05 was considered statistically significant.

## 3 Results

### 3.1 The relationship between FGF21 expression in human PDAC tissues and invasion and metastasis

**Figure 1:**
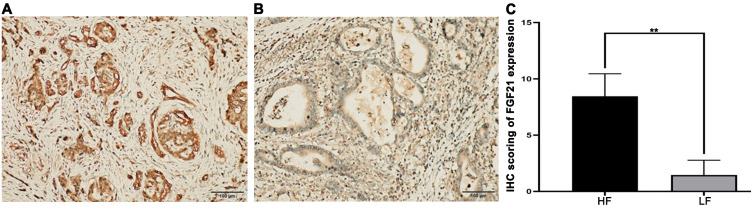
FGF21 expression in PDAC tissues by immunohistochemistry (×200). **(A)** Representative images of high expression of FGF21. **(B)** Representative images of low expression of FGF21. **(C)** Comparison of Immunohistochemical (IHC) scoring between groups of FGF21 high-expression (HF) and low-expression (LF) in PDAC tissue specimens (primary pancreatic ductal adenocarcinoma). Results were expressed as mean ± SD. T-tests was used to assess significance. **P<0.01.

Immunohistochemical staining and scoring of FGF21 expression were performed on PDAC tissue specimens from 72 patients. The results showed that 22 cases (30.56%) had high expression of FGF21 (++ to +++), while 50 cases (69.44%) had low expression (− to +) (**Figure 1**), with a significant difference between the two groups (*P*<0.01). Further statistical analysis revealed in the **Table 2**. It showed that a negative correlation exists between TNM stages and FGF21 expression levels, where lower FGF21 expression in PDAC predicts higher tumor stages. Furthermore, the PDAC patients with lymph node metastasis and vascular invasion have a higher proportion of low expression of FGF21. However, no significant difference was observed in the proportion of low FGF21 expression between patients with and without perineural invasion. In patients with the 3-year postoperative follow-up 42 cases (80.77%) in the low expression group had recurrence, while only 10 cases (40.00%) in the high expression group had recurrence, with a significant difference between the two groups (*P*<0.05), indicating that patients with low expression of FGF21 had a higher tumor recurrence rate. Taken together, these results strongly suggest that loss of FGF21 expression may contribute to a more aggressive and metastatic phenotype in pancreatic cancer.

**Table 2:** The correlation between FGF21 expression in human pancreatic cancer tissues and invasion and metastasis.

	Low expression of FGF21	High expression of FGF21	The rate of low FGF21 expression	P*
TNM staging				
Stage I-II	10	16	10/26	0.0064
Stage III-IV	40	6	40/46	
Lymph node metastasis				
+	36	2	36/38	0.0008
−	14	20	14/34	
Vascular invasion				
+	32	4	32/36	0.0275
−	18	18	18/36	
Nerve invasion				
+	40	14	40/54	0.4088
−	10	8	10/18	
3-year postoperative follow-up				
Recurrence	42	10	42/52	0.0390
No recurrence	8	12	16/20	

*The chi-square test was used for statistical analysis, and Fisher’s exact test was applied when expected frequencies were less than 5. Differences were considered to be significant at *p* values< 0.05.

### 3.2 The relationship between FGF21 expression in human PDAC tissues and the tumor microenvironment of PDAC

**Figure 2:**
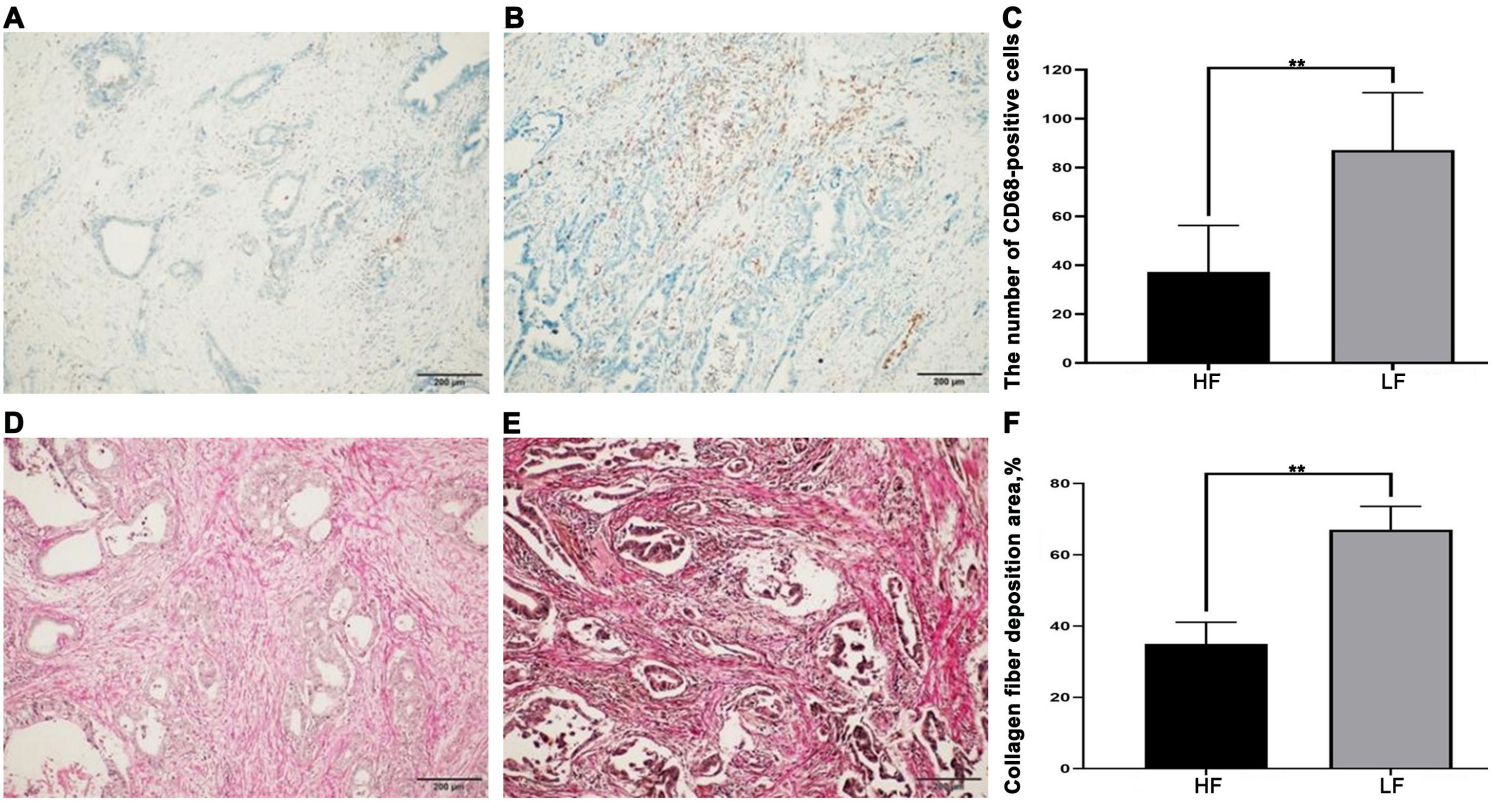
CD68 expression and collagen fiber deposition in human PDAC tissues (×200). **(A)** Representative images of CD68-positive cells in the HF (sparsely distributed in the tumor microenvironment). **(B)** Representative images of CD68-positive cells in the LF (densely distributed in the tumor microenvironment). **(C)** Comparison of CD68-positive cells between groups. **(D)** Representative images of collagen fiber deposition in the HF (in the tumor microenvironment). **(E)** Representative images of collagen fiber deposition in the LF (in the tumor microenvironment). **(F)** Comparison of collagen fiber deposition between groups. Results were expressed as mean ± SD (HF- FGF21 high-expression group, LF- FGF21 low-expression group). T-tests was used to assess significance. **P<0.01.

In the tumor microenvironment, CD68 immunohistochemical positivity indicates macrophages (**Figures 2A, B**). The results showed that the number of infiltrating macrophages in the FGF21 high-expression group was 37.27 ± 18.97, while it was 87.08 ± 23.00 in the FGF21 low-expression group. There was a significant difference between these two groups (*P*<0.01, **Figure 2C**).

Sirius Red staining was used to detect collagen fiber deposition in the tumor microenvironment. The results showed (**Figures 2D, E**) that compared to the FGF21 high-expression group, the FGF21 low-expression group in PDAC had a significant amount of collagen fiber deposition in the stroma. The difference between the two groups was statistically significant (*P*<0.01, **Figure 2F**). The heightened macrophage infiltration and collagen deposition observed in FGF21-low tumors indicate a more pronounced inflammatory response within the tumor microenvironment.

### 3.3 The inhibitory effect of FGF21 on the invasion and migration of PDAC cell lines *in vitro*

**Figure 3:**
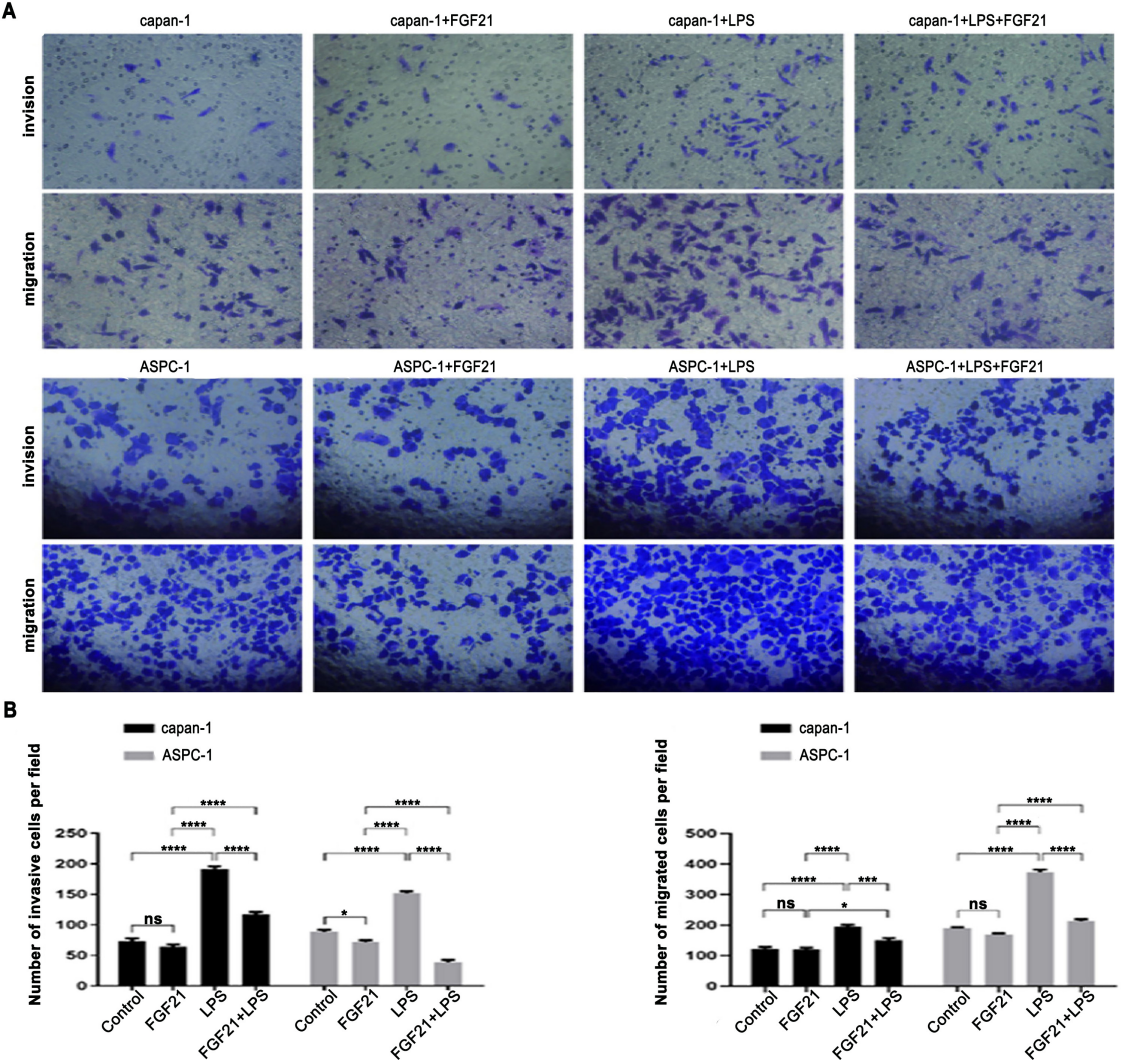
Inhibitory effect of FGF21 on the invasion and migration of PDAC cell lines *in vitro*. **(A)** Representative images of capan-1 and ASPC-1 in Transwell assays (×200); **(B)** Comparison the number of invasive cells (left), and migrated cells (right) between groups. Results are expressed as the mean ± SD from three independent samples (LPS- Lipopolysaccharide). ANOVA was used to assess significance. *P<0.05, *** *P*<0.001, ****P<0.0001, ns, no significance.

The tumor microenvironment of PDAC promotes tumor progression by activating inflammatory signaling pathways. Given the significant negative correlation observed between levels of FGF21 and stromal inflammation in human PDAC, we stimulated capan-1 and ASPC-1 cells using LPS to induce inflammatory signaling. As shown (**Figures 3A, B**), no significant difference was observed in the number of invasive and migratory cells between the FGF21-treated group and the control group. However, compared to the control group, stimulation of LPS significantly increased the number of invasive and migratory cells. Importantly, co-treatment with LPS and FGF21 significantly attenuated this increase (*P*<0.05). These results demonstrate that FGF21 effectively inhibits the invasion and migration of PDAC cell lines under inflammatory conditions.

### 3.4 The inhibitory effect of FGF21 on the metastasis of PDAC cell lines *in vivo*

**Figure 4:**
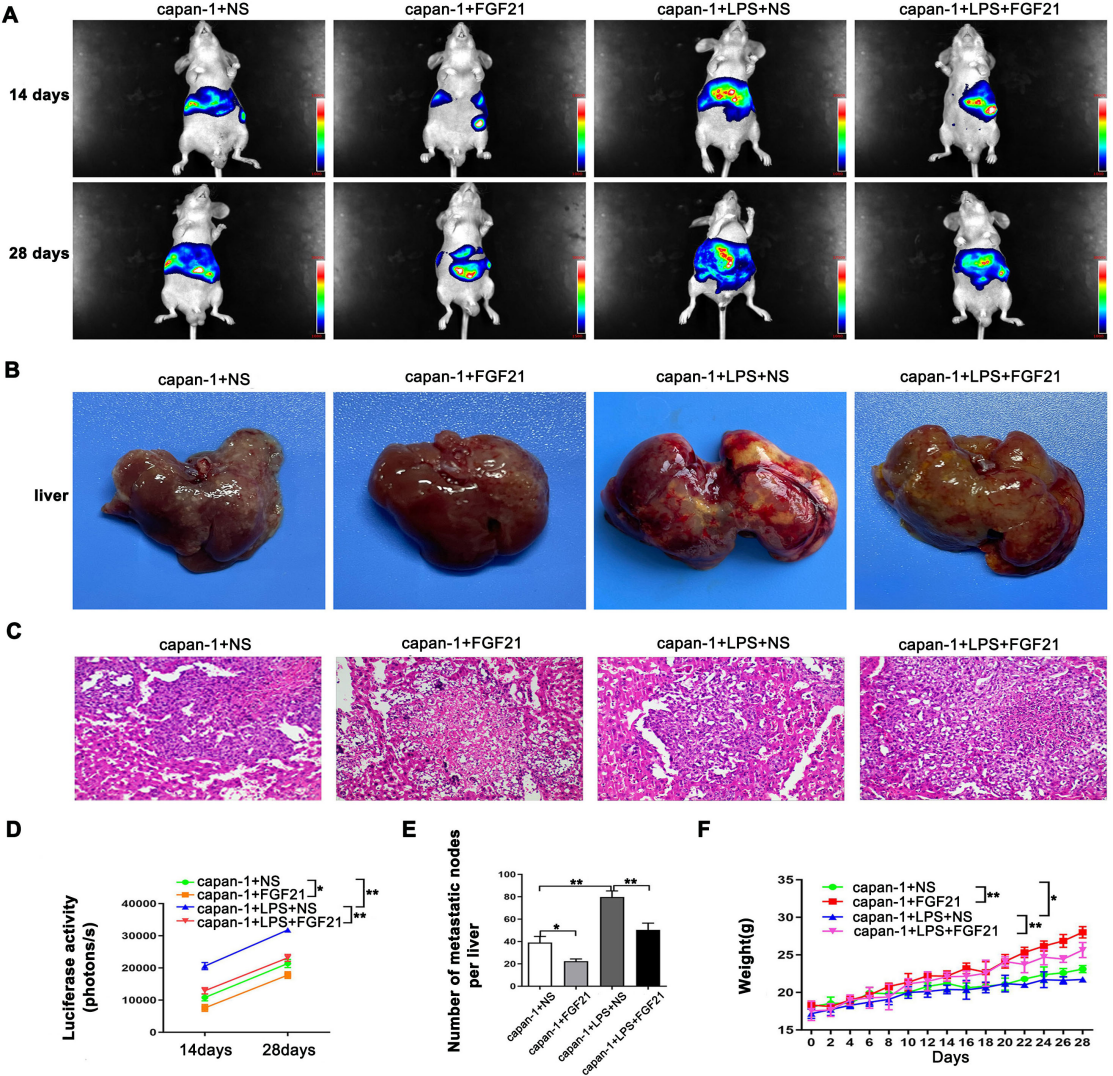
Inhibitory effect of FGF21 on the metastasis of PDAC cell lines *in vivo*. **(A)** Representative images of liver metastasis nodules by fluorescence. **(B)** Gross appearance of liver metastasis nodules. **(C)** Microscopic appearance of liver metastasis nodules. **(D)** Comparison of imaging fluorescence intensity between groups. **(E)** Comparison the number of liver metastasis nodules between groups. **(F)** Comparison of mouse body weight between groups. Results are presented as the mean ± SD from three independent mice per group (NS-normal saline). ANOVA was used to assess significance. *P<0.05, **P<0.01.

Compared to *in vitro* models, the mouse spleen-liver metastasis model better recapitulates key features of the human pancreatic cancer microenvironment. As shown (**Figures 4A, D, F**), both intensity of fluorescence and body weight of mouse increased significantly over time (*P*<0.05), indicating that the tumor burden in the animals progressively increased. Relative to the control group, the LPS group significantly increased liver metastasis, while the FGF21-treated markedly attenuated this LPS-induced effect (*P*<0.05). Simultaneously, we observed consistent changes in the extracted liver tissues of the mice (**Figures 4B, E**). H&E staining of the metastatic nodules revealed that within the normal liver tissue, atypical cells were arranged in nests or gland-like patterns, with numerous mitotic figures and accompanying tissue necrosis (**Figure 4C**). These *in vivo* results demonstrate that FGF21 significantly reduces the liver metastatic potential of PDAC cells.

### 3.5 Transcriptome sequencing analysis of PDAC cell lines after FGF21 treatment

**Figure 5:**
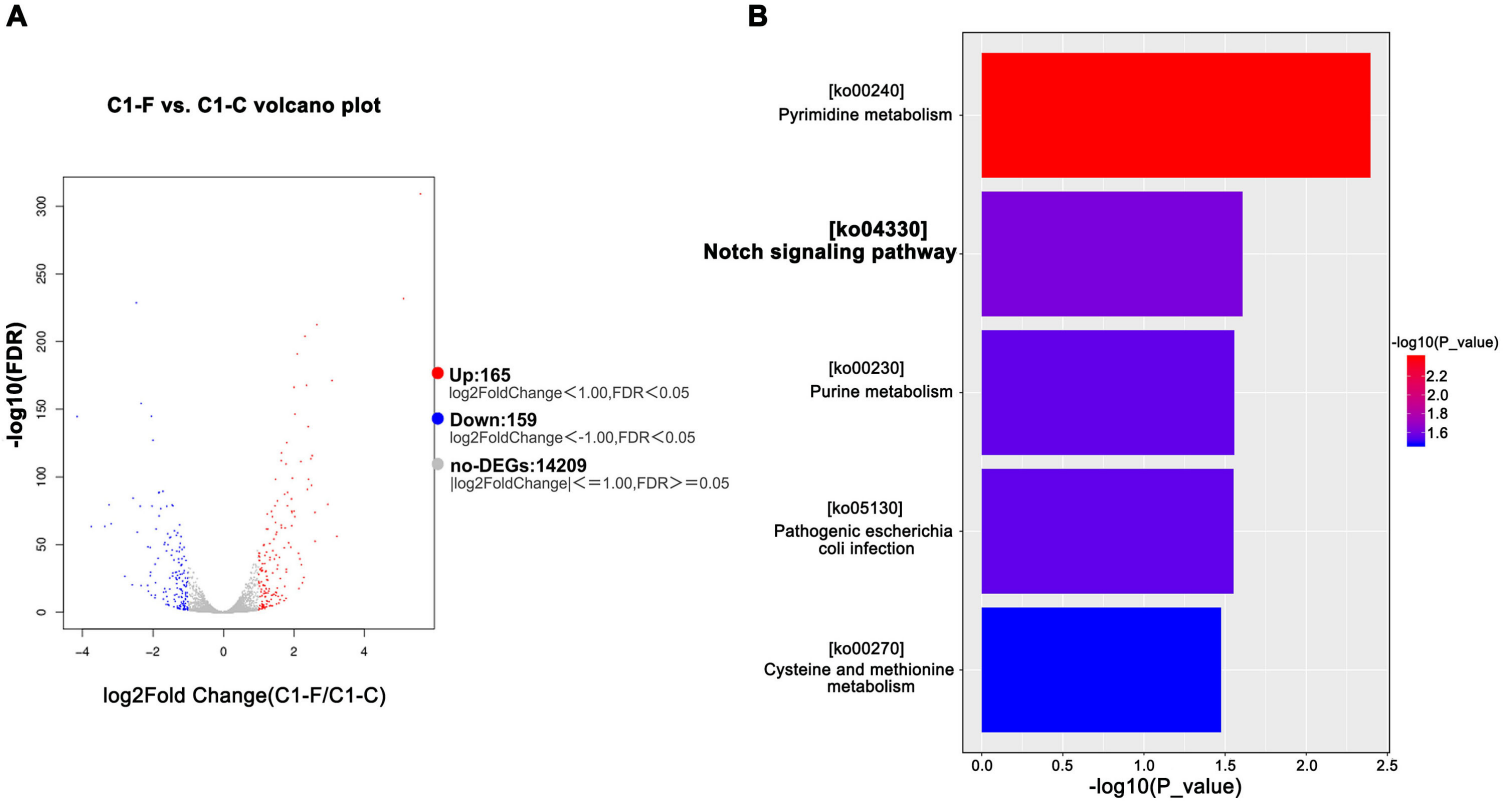
Transcriptome analysis of PDAC cell lines after FGF21 treatment. **(A)** Significantly differentially expressed genes after FGF21 treatment. **(B)** KEGG enrichment results of differentially expressed genes.

To further investigate the mechanism underlying FGF21-mediated inhibition of PDAC cell invasion and metastasis, we conducted transcriptome sequencing analysis on FGF21-treated cells. Compared to the control group, the FGF21-treated group induced differential expression of 352 genes: 165 upregulated and 159 downregulated (**Figure 5A**). Significantly downregulated genes included RBPJ, HES2, TLR4, and ZEB1, etc. KEGG enrichment analysis revealed significant enrichment of differentially expressed genes in the Notch signaling pathway (**Figure 5B**). These transcriptomic data suggest that FGF21 exerts its effects, at least in part, by suppressing the Notch signaling pathway, a key regulator of inflammation and tumor progression.

### 3.6 The inhibitory effect on the Notch signaling pathway in PDAC cell lines by FGF21

**Figure 6:**
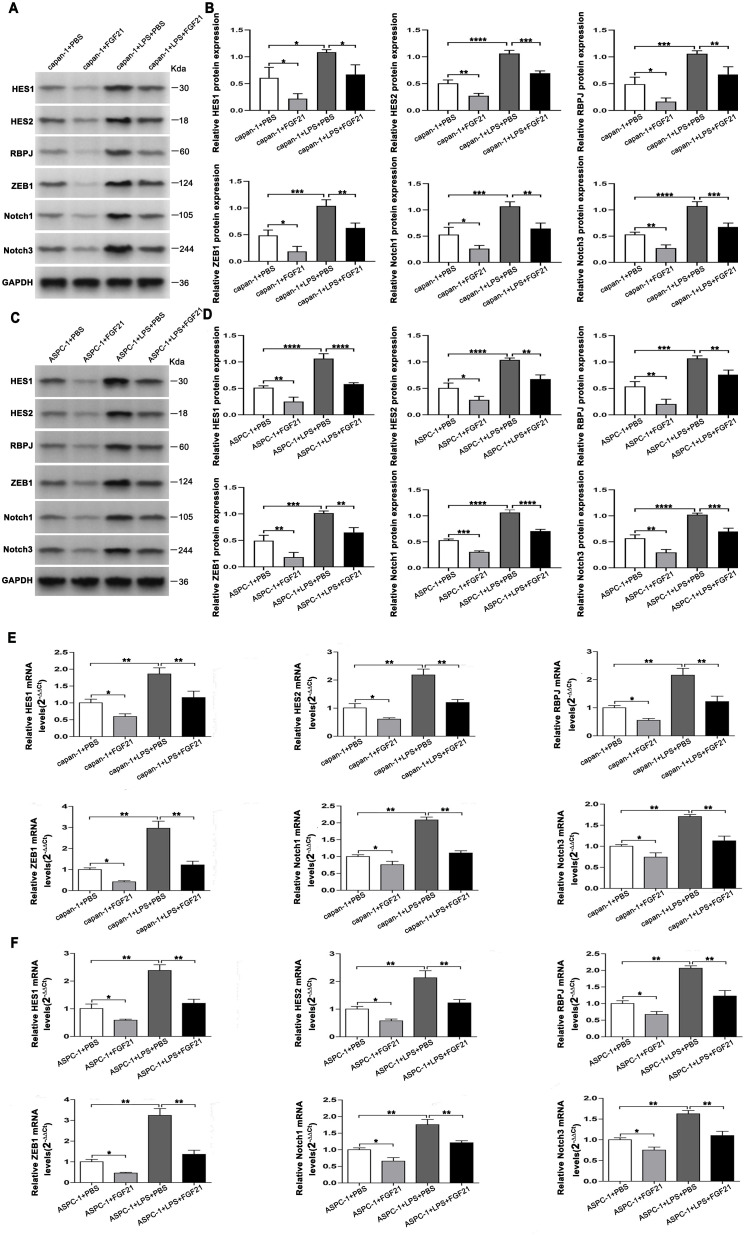
Inhibition on the Notch signaling pathway in PDAC cell lines by FGF21. **(A)** Protein expression levels were assessed by immunoblotting in capan-1 cells. **(B)** Quantifying the Western blot bands in capan-1 cells (The expression was normalized to *GAPDH*). **(C)** Protein expression levels were assessed by immunoblotting in ASPC-1 cells. **(D)** Quantifying the Western blot bands in ASPC-1 cells (The expression was normalized to *GAPDH*). **(E)** mRNA expression levels in capan-1 cells (Data are normalized to the levels of capan-1+PBS). **(F)** mRNA expression levels in ASPC-1 cells (Data are normalized to the levels of ASPC-1+PBS). Results are presented as the mean ± SD from three independent samples (PBS- Phosphate-Buffered Saline). ANOVA was used to assess significance. Notch signaling components (*HES1, HES2, RBPJ, ZEB1, Notch1, Notch3*) and loading control (*GAPDH*). *P<0.05, **P<0.01, ***P<0.001, ****P<0.0001.

Based on transcriptome sequencing results, we further validated the effect of FGF21 on the Notch signaling pathway in PDAC cell lines. Western Blot and Reverse Transcription Quantitative Real-Time PCR(RT-PCR) analyses revealed that the FGF21-treated group significantly reduced key Notch signaling components including TRL4, HES1, HES2, RBPJ, Notch1, and Notch3 in both capan-1 and ASPC-1 compared to the control group (**Figure 6**). Furthermore, FGF21 significantly attenuated LPS-induced activation of the Notch pathway in these cells relative to the LPS-only group (*P*<0.05). These results confirm that FGF21 suppresses the Notch pathway, thereby counteracting its inflammation-driven activation and contributing to the inhibition of PDAC progression.

### 3.7 The inhibitory effect of FGF21 on IL-17A expression in PDAC cell lines

**Figure 7:**
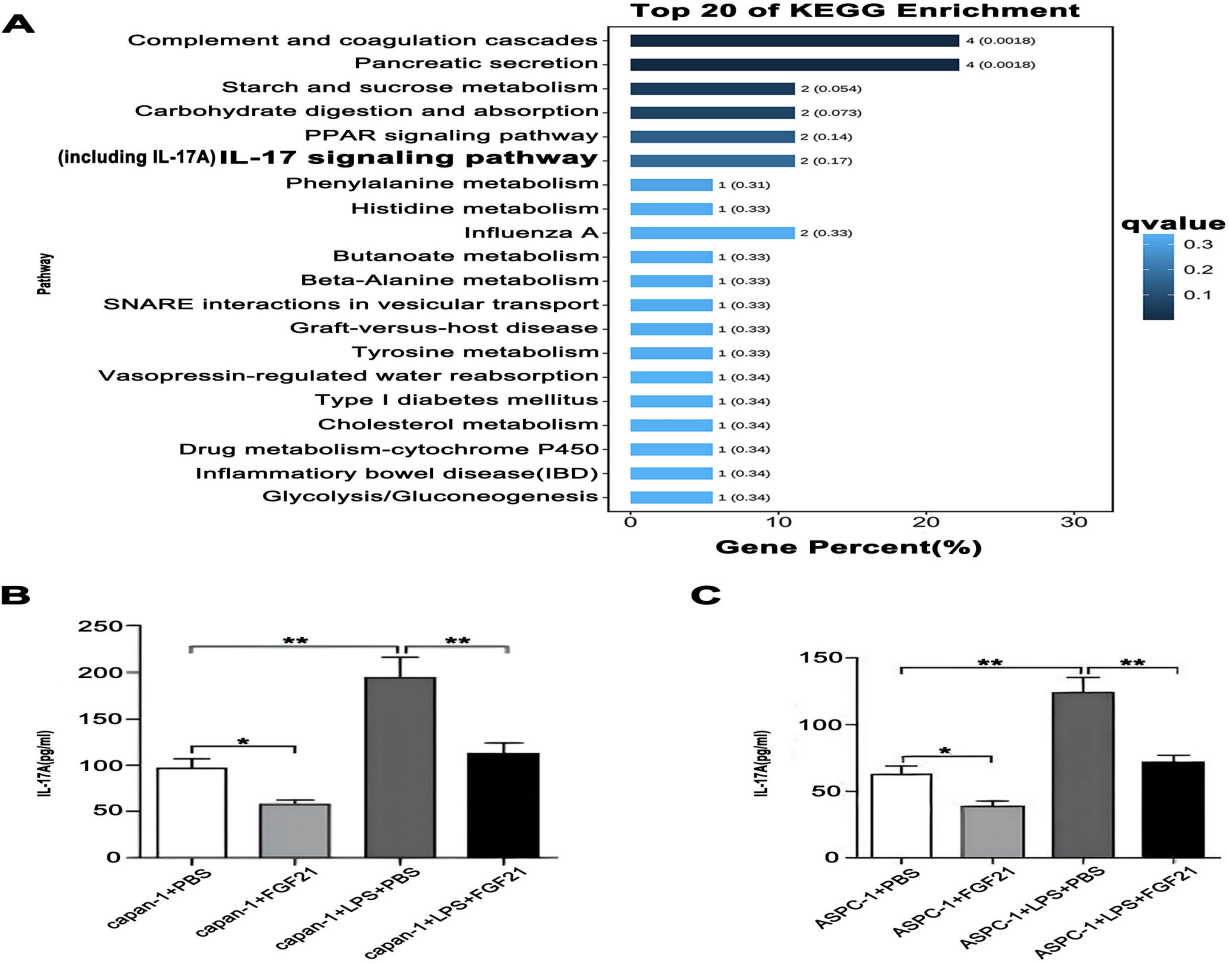
The inhibitory effect of FGF21 on IL-17A in PDAC cell lines. **(A)** KEGG enrichment analysis of FGF21-related genes, revealed significant enrichment in the IL-17. **(B)** IL-17A concentration in the culture medium of capan-1 after FGF21 treatment. **(C)** IL-17A concentration in the culture medium of ASPC-1 after FGF21 treatment. Results are presented as mean ± SD from three independent samples. ANOVA was used to assess significance. *P<0.05, **P<0.01.

KEGG pathway enrichment analysis of FGF21-associated genes was performed using the R package clusterProfiler (v4.0). Candidate gene overexpression in KEGG pathways was assessed via hypergeometric testing with Benjamini-Hochberg correction for multiple comparisons. Bubble plot analysis of the top 20 enriched pathways revealed significant enrichment in the IL-17 (FDR< 0.3; **Figure 7A**). Subsequently, we detected the levels of IL-17A in the culture medium of capan-1 and ASPC-1 treated with LPS and/or FGF21 using ELISA kits. The results showed that compared with the control group and LPS+PBS group, the FGF21 treatment significantly reduced the IL-17A levels in the culture medium of PDAC cell lines and suppressed the LPS-induced increase in IL-17A levels in the culture medium (**Figures 7B, C**, *P*<0.05). Based on the bioinformatic prediction and subsequent experimental validation, we conclude that the suppression of IL-17 signaling represents a key anti-inflammatory mechanism through which FGF21 exerts its inhibitory effects in PDAC.

### 3.8 FGF21 downregulates the Notch signaling pathway through IL-17A

**Figure 8:**
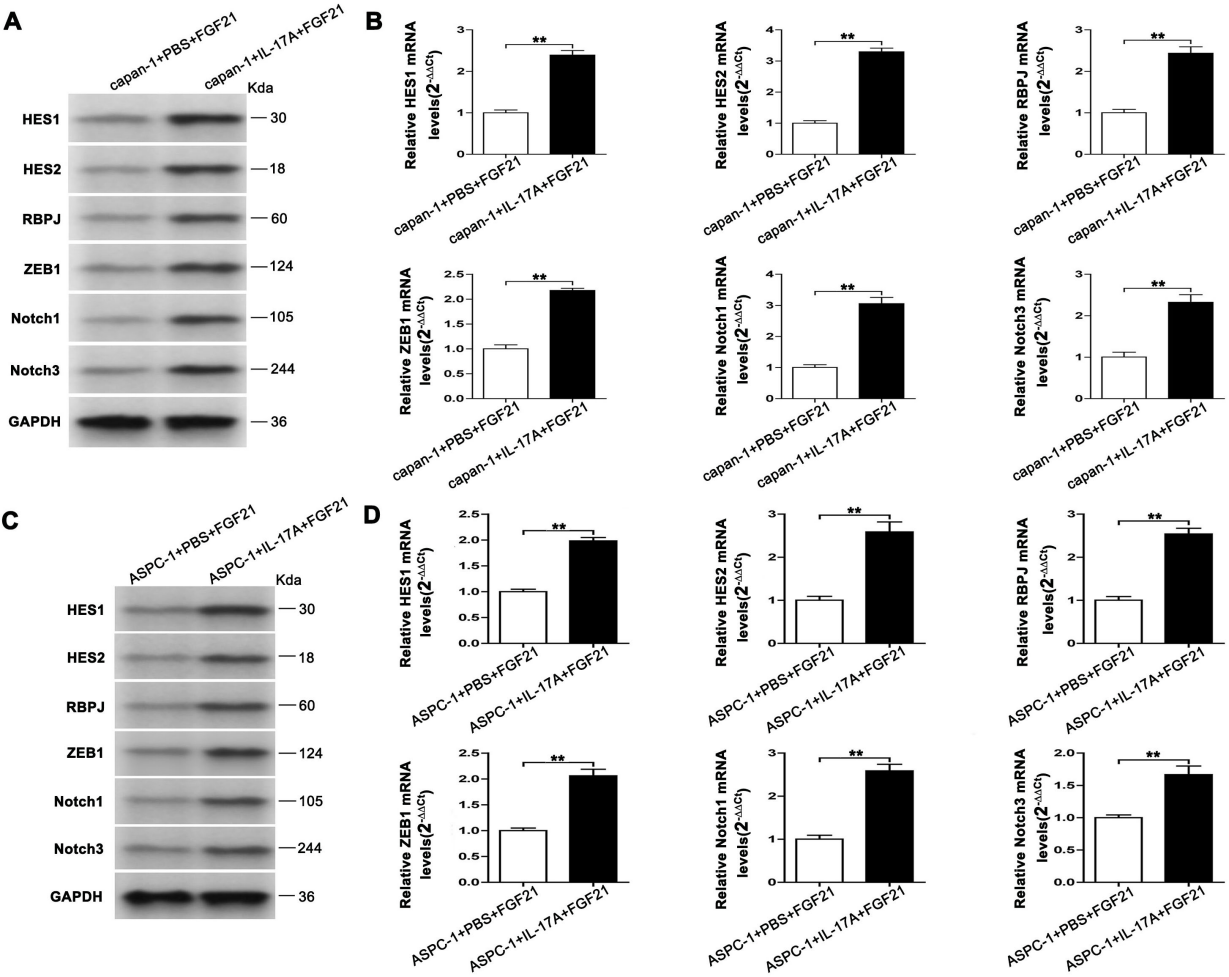
FGF21 downregulates the Notch signaling pathway through IL-17A in cells. **(A)** Protein expression levels were assessed by immunoblotting in capan-1 cells. **(B)** mRNA expression levels in capan-1 cells (Data are normalized to the levels of capan-1+PBS+FGF21). **(C)** Protein expression levels were assessed by immunoblotting in ASPC-1 cells. **(D)** mRNA expression levels in ASPC-1 cells (Data are normalized to the levels of ASPC-1+PBS+FGF21). Results are presented as the mean ± SD from three independent samples. T-tests were used to assess significance. Notch signaling components (*HES1, HES2, RBPJ, ZEB1, Notch1, Notch3*) and loading control (*GAPDH*). **P<0.01.

It is indicated that in PDAC, IL-17A can activate the Notch signaling pathway and synergize with it to collaboratively promote the invasion and metastasis of PDAC. To further investigate the upstream-downstream relationship between IL-17A and the Notch signaling pathway, we divided capan-1 and ASPC-1 cells into two groups: FGF21 + IL-17A group and FGF21 + PBS group. Exogenous IL-17A (200 ng/ml) or an equal volume of PBS was added to the culture medium of the respective groups and incubated for 24 hours. The activation of the Notch signaling pathway in the cells was then assessed at both the mRNA and protein level using RT-PCR and Western blotting. The results showed that, compared with the FGF21+PBS group, both the mRNA and protein expression levels of HES1, HES2, RBPJ, ZEB1, Notch1, and Notch3 were significantly increased in the FGF21+IL-17A group of capan-1 and ASPC-1 cells (**Figure 8**, *P*<0.01). We conclude that FGF21 inhibits PDAC invasion and metastasis primarily by disrupting the activation of the Notch pathway by its upstream regulator, IL-17A.

## 4 Discussion

Fibroblast Growth Factor 21 (FGF21), known for its anti-inflammatory and metabolic regulatory properties, is increasingly recognized for its potential role in cancer. While clinical development has focused on metabolic diseases such as diabetes and NAFLD, emerging evidence indicates that FGF21 also exerts antitumor effects in cancers including hepatocellular and prostate carcinoma, potentially through modulating autophagy, glycolysis, and ferroptosis (22–26).

In the pancreas, FGF21 is abundantly expressed under normal conditions but is markedly downregulated during pancreatitis and pancreatic ductal adenocarcinoma (PDAC) (10). Notably, exogenous FGF21 ameliorates pancreatic inflammation (11, 27), suggesting a protective role. Our initial findings revealed reduced FGF21 expression in PDAC tissues, which correlated with advanced tumor stage, lymph node metastasis, vascular invasion, and poor prognosis, implicating FGF21 loss in PDAC aggressiveness.

Previously, we showed that FGF21 administration in Kras^G12D^/+ mice on a high-fat diet reduced inflammatory cell infiltration and fibrosis (12). Here, we demonstrated that low FGF21 expression in human PDAC correlates with increased macrophage infiltration and stromal fibrosis. Since macrophages promote PDAC progression by secreting inflammatory mediators and activating stellate cells (19, 28, 29), these results suggest that FGF21 may inhibit metastasis by attenuating inflammation and desmoplasia. We hypothesized that FGF21 suppresses invasion and metastasis by modulating the tumor microenvironment—a key driver of PDAC progression.

Functional assays supported this notion: FGF21 significantly reduced the migration and invasion of LPS-stimulated PDAC cells (Capan-1 and ASPC-1). In a splenic injection model of liver metastasis, LPS promoted metastatic nodule formation, an effect counteracted by FGF21 co-administration.

Mechanistically, transcriptome analysis revealed that FGF21 treatment significantly altered the Notch signaling pathway. LPS robustly activated Notch components (Notch1, Notch3, Hes1, Hes2, ZEB1, RBPJ) in PDAC cells, while FGF21 effectively suppressed this activation. Given the established role of Notch signaling in PDAC invasion, EMT, and stromal remodeling (30–38), our data suggest that FGF21 inhibits metastasis through Notch pathway repression.

We further identified IL-17A as a key mediator linking FGF21 to Notch signaling, IL-17A is a pro-inflammatory cytokine that promotes PDAC progression (39–45). TCGA analysis indicated a strong association between FGF21 and IL-17A in PDAC. Mirroring its effects in liver models, FGF21 inhibited IL-17A expression in LPS-stimulated PDAC cells and in mouse PDAC tissues (46). Since IL-17A stimulates Notch activation via NF-κB (47), we propose that FGF21 downregulates IL-17A, thereby attenuating Notch signaling.

In summary, FGF21 expression is reduced in PDAC and correlates with improved patient outcomes. Functionally, FGF21 constrains invasion and metastasis by targeting the IL-17A–Notch axis, thereby disrupting a critical inflammatory-metastatic circuit in PDAC. Our findings nominate FGF21-based therapy as a promising strategy for mitigating metastasis in this inflammatory malignancy.

While our study reveals an interesting biological role for FGF21, several key limitations remain. The *in vivo* findings require validation in larger cohorts. Mechanistic links to IL-17A and NF-κB are still correlative and need definitive proof. The influence of FGF21 on the tumor stroma is also unknown. Ultimately, the leap to human disease necessitates validation in models that capture the heterogeneity of human pancreatic cancer. Thus, while our findings reveal a promising therapeutic avenue, their translation mandates deeper mechanistic exploration and robust validation.

## Data Availability

Original datasets are available in a publicly accessible repository: The original contributions presented in the study are publicly available. This data can be found here: https://data.mendeley.com/datasets/n6szx9594h/2.
